# Survival of Laryngeal Cancer Patients Depending on Zinc Serum Level and Oxidative Stress Genotypes

**DOI:** 10.3390/biom11060865

**Published:** 2021-06-10

**Authors:** Jakub Lubiński, Ewa Jaworowska, Róża Derkacz, Wojciech Marciniak, Katarzyna Białkowska, Piotr Baszuk, Rodney J. Scott, Jan A. Lubiński

**Affiliations:** 1 Clinic of Otolaryngology, Pomeranian Medical University, Unii Lubelskiej 1, 71-252 Szczecin, Poland; jaworowska@tlen.pl; 2 ReadGene, Alabastrowa 8, 72-003 Grzepnica, Poland; roza.derkacz@gmail.com (R.D.); wojciech.marciniak90@gmail.com (W.M.); lubinski@pum.edu.pl (J.A.L.); 3 Department of Genetics and Pathology, International Hereditary Cancer Centre, Pomeranian Medical University, Unii Lubelskiej 1, 71-252 Szczecin, Poland; katarzyna.bialkowska@pum.edu.pl (K.B.); baszukpiotr@gmail.com (P.B.); 4 Discipline of Medical Genetics, Faculty of Health, School of Biomedical Sciences, University of Newcastle, Callaghan, NSW 2308, Australia; rodney.scott@newcastle.edu.au; 5 The Hunter Medical Research Institute, Kookaburra Cct, New Lambton Heights, NSW 2305, Australia

**Keywords:** laryngeal cancer, zinc, antioxidant enzymes

## Abstract

Stress contributes to various aspects of malignancy and could influence survival in laryngeal cancer patients. Among antioxidant mechanisms, zinc and the antioxidant enzymes superoxide dismutase 2, catalase and glutathione peroxidase 1 play a major role. The aim of this study was a prospective evaluation of the survival of patients with laryngeal cancer in relation to serum levels of zinc in combination with functional genotype differences of three key antioxidant enzymes. The study group consisted of 300 patients treated surgically for laryngeal cancer. Serum zinc levels and common polymorphisms in *SOD2*, *CAT* and *GPX1* were analyzed. The risk of death in patients with the lowest zinc levels was increased in comparison with patients with the highest levels. Polymorphisms of antioxidant genes by themselves were not correlated with survival, however, serum zinc level impact on survival was stronger for *SOD2 TC/TT* and *CAT CC* variants. *GPX1* polymorphisms did not correlate with zinc levels regarding survival. In conclusion, serum zinc concentration appears to be an important prognostic factor for survival of patients diagnosed with laryngeal cancer. When higher zinc levels were correlated with polymorphisms in *SOD2* and *CAT* a further increase in survival was observed.

## 1. Introduction

Laryngeal cancer represents one of the most common head and neck malignancies, accounting for approximately 20% of all cases, and up to 40% of patients present with advanced disease at the time of diagnosis [1,2,3]. The proper treatment for locally advanced laryngeal cancer includes surgery, chemotherapy, radiotherapy, or some combination of these. Regardless of the treatment option, survival in laryngeal cancer patients is poor with 50–70% 5-year overall survival rates [4]. Given the dismal outcomes for this disease, which have not changed for decades, the identification of modifiable factors, including diet and lifestyle, that could improve prognosis of this fatal disease is necessary. One avenue that warrants interrogation, as it is likely to influence survival, is the role of anti-oxidants in protection from free radicals and how variation in genes involved in this process is associated with disease outcomes.

There are a growing number of studies that have reported that reactive oxygen species (ROS) contribute to various aspects of malignancy, including carcinogenesis, aberrant growth, metastasis, and angiogenesis [5]. ROS are chemically reactive molecules containing oxygen, which are generated in cells by certain organelles such as mitochondria or by external factors such as ionizing radiation [6]. ROS are capable of causing lipid peroxidation, altering the activity of antioxidant enzymes which culminate in DNA damage and subsequently genomic instability [7,8]. ROS-mediated damage to cellular macromolecules results in deleterious effects associated with carcinogenesis [9,10]. The production of ROS and the antioxidant mechanisms remain in dynamic equilibrium such that when ROS levels exceed the antioxidant cell capacity, oxidative stress occurs [11,12]. Antioxidant protection is divided into non-enzymatic and enzymatic mechanisms. The non-enzymatic antioxidants are represented by molecules characterized by the ability to rapidly inactivate free radicals and oxidants [13]. Among them are metal binding proteins, glutathione, uric acid, melatonin, bilirubin, polyamines, vitamins A, E, C, ceruloplasmin and trace metals, e.g., selenium, zinc, copper, iron and manganese [14]. We have previously reported on the influence of serum selenium levels and survival in laryngeal cancer patients [15]. A prospective study of 296 patients undergoing treatment for laryngeal cancer showed that selenium levels in excess of 70 µg/L were associated with improved outcome. This promising result suggested that a correlation between survival in laryngeal cancer patients and the levels of other trace metals involved in antioxidant activity should be investigated.

Zinc is an essential element for human metabolism and is required for multiple biological functions. Zinc is involved in immune function, wound healing, blood clotting, thyroid function and is required for more than 300 different enzymes [16]. Zinc deficiency, which causes increased oxidative stress, leads to a wide range of metabolic diseases, endocrine disorders, developmental imbalances, neurodegenerative problems, immune deficiencies, inflammatory pathologies, skin diseases and wound healing [17,18]. Many reports exist between zinc levels and cancer occurrence but to our knowledge little has been reported on disease progression. There have been no studies specifically evaluating the role of circulating zinc and disease prognosis following a diagnosis of laryngeal cancer. Thus, the effect on progression of laryngeal cancer remains unknown.

Enzymatic antioxidant mechanisms consist of an interacting network of antioxidant enzymes, that include superoxide dismutases (*SOD*), glutathione peroxidases (*GPX*) and catalases (*CAT*) [19]. These enzymes break down reactive compounds into oxygen and water, thereby removing free radical damage to a cell. *SOD* is represented in three forms: *SOD1*, which is located in the cytoplasm; *SOD2*, which exists in the mitochondria; and *SOD*3, which functions in the extracellular matrix. Of these isoforms, *SOD2*, encoded by the nuclear gene located on chromosome 6q25.3, was found to modify cancer susceptibility, largely due to several functional single nucleotide polymorphisms [20]. The most studied polymorphism is *SOD2* 47T/C (rs4880), or Val16Ala, which results in the incorporation of either alanine (C allele) or valine (T allele) in the mitochondrial targeting sequence on the 16th amino acid.

Glutathione peroxidases constitute another major family of antioxidant enzymes. Among eight *GPX*s expressed in humans, *GPX1* is the most abundant and ubiquitous isoform [21]. A polymorphism (rs1050450) *GPX1*c.599T>C results in the amino acid substitution of leucine (T variant) for proline (C variant) at position 198, which results in a decrease in the activity of *GPX1* [22,23].

Catalase (*CAT*) is an important enzyme which can neutralize reactive oxygen species by converting H_2_O_2_ into H_2_O and O_2_ [24]. A common polymorphism has been identified in the prompter region of *CAT c.*262C>T, rs1001179, which influences transcription factor binding, altering basal transcriptional activity and subsequent expression of this enzyme. Compared with the wild-type allele, the variant T allele has been linked to lower enzyme activity and increased levels of ROS [25,26].

The goal of the current study was to evaluate whether circulating zinc levels, at the time of diagnosis, and polymorphisms in *SOD2, CAT* and *GPX1* are associated with survival among laryngeal cancer patients.

## 2. Material and Methods

The current study includes 300 patients treated surgically in the period from July 2009 to February 2017 due to squamous cell carcinoma of the larynx at one University Hospital in Szczecin, Poland within the Pomeranian Medical University. The Otolaryngology Clinic at the University Hospital surgically treats more than 90% of patients diagnosed with laryngeal cancer in the West Pomeranian region of Poland. The study group accounted for over 70% of all patients treated for laryngeal cancer in the study period. A pathologic diagnosis of squamous cell laryngeal cancer was confirmed by histopathological review of postoperative material at a single central pathology laboratory in Szczecin. The study was approved by the Ethics Committee of the Pomeranian University of Medicine in Szczecin (KB-0012/167/13; 18 November 2013). All eligible patients provided written informed consent for a blood draw specifically for research purposes and for storage of the blood sample in an existing research biobank. Consenting patients were asked to fast for at least four hours prior to blood collection. A blood sample (10 mL) was obtained during the diagnostic workup between 8 a.m. and 2 p.m. and was processed within 30 and 120 min of collection to separate the serum from the cellular fraction. The serum samples were stored at −80 °C. In November 2017 information on deaths which appeared during the prospective observation period was collected and obtained from the Department of State Infrastructure of the Ministry of Digital Affairs. Death was all-cause mortality because the specific cause of death was not available. The average period of observation for living patients was 62.2 months (min–10, max–101).

Serum zinc levels were measured in the READ-GENE S.A. laboratory using the technique of inductively coupled mass spectroscopy (ICP-MS). Zinc (Zn^65^) concentration in serum samples was measured by inductively coupled plasma mass spectroscopy (ICP-MS) technique using a NexION 350D (PerkinElmer, Shelton, CT, USA) instrument. Zinc was measured in DRC mode with methane (CH_4_, AirProducts, purity > 0.999) as a reaction gas for removing spectral interferences. To compensate for instrument drift and matrix effects, germanium was set as internal standard. All the reagents were prepared fresh every day. Blank reagent consisted of 0.65% HNO_3_ Suprapur Grade (Merck, Darmstadt, Germany), 0.002% Triton X-100 (PerkinElmer, Shelton, CT, USA) was prepared. Calibration curve standards (100; 200; 500; 1000 and 1500 µg/L) were prepared by diluting stock solution of 10 mg/L Multi-element Calibration Standard 3 (PerkinElmer Pure Plus, Shelton, CT, USA) with blank reagent. An external calibration method was used. The correlation coefficient of the Zn calibration curve was always greater than 0.999. Prior to analysis, serum samples were thawed and centrifuged (7000× *g* rpm, 15 min) and the supernatant was diluted 100 times with blank reagent and directly measured. ClinChek^®^ Serum Control Level I (Recipe, Munich, Germany) was used as a reference material.

Genotypes of antioxidant enzymes genes were established using real-time PCR with TaqMan probes. The following polymorphism were studied: *GPX1* 599C/T, rs1050450, Pro198Leu–proline (C variant) replaced with leucine (T variant); *SOD2* 47T/C, rs4880, Val16Ala–valine (T variant) replaced with alanine (C variant) and *CAT* 262C/T, rs1001179-variant T allele, which is associated with lower enzyme activity. Polymorphisms in *SOD2* and *CAT* were analyzed using predesigned TaqMan assays and for *GPX1* a customized assay was used (primer F: CATTGACATCGAGCCTGACATC; primer R: CACTGCAACTGCCAAGCA; Vic probe: CAGCTGGGCCCTTG; Fam probe: CAGCTGAGCCCTTG). The reaction mix for each sample consisted of TaqMan Genotyping Assay x 40 (Applied Biosystems, Warrington, UK), GoTaq^®^ Probe qPCR Master Mix (Promega, Madison, CA, USA), and deionized water in accordance with the TaqMan Assay manufacturer’s instructions. DNA samples and reaction mix were placed in 384-well plates (Axygen, Union City, CA, USA), then real-time PCR reactions were performed on LightCycler^®^ 480 (Real-Time PCR System, Roche Diagnostics, Rotkreuz, Switzerland). For genotyping data analysis, LightCycler480 Basic Software Version 1.5 was used (Software release 1.5.0.1.62 SP2; Roche Diagnostics, 2012, USA).

Statistical analysis. Treatment outcomes were evaluated based on survival time and the number of deaths that occurred during the observation period. The entire group was divided into three subgroups of similar size depending on zinc serum levels. The range of zinc levels determined in this way were used to define subgroups of patients depending on the polymorphisms of oxidative stress enzymes. The relationship between the number of deaths, survival time and the levels of serum zinc was statistically estimated using multivariable Cox proportional hazard models. Multivariable analyses took into account the influence of age, sex, clinical stage, chemotherapy, radiotherapy and pack-years of smoking.

## 3. Results

The characteristics of the 300 laryngeal cancer patients are presented in Table 1. The mean age of diagnosis was 61 years (range 41 to 82 years). The majority of patients were male (85%), 96% had a history of smoking and 42% were diagnosed with stage IV disease. A total of 45% of patients received postoperative radiotherapy and 10% received chemotherapy. A total of 116 patients died during the follow-up period. The average survival time for those who died during the study was 26 months and for the entire cohort was 47.6 months. Overall, the mean serum zinc level was 640.43 μg/L (range 357.76 µg/L to 1317.87 µg/L) for all participants.

Zinc serum levels appeared to influence survival. Table 2 demonstrates a statistically significant increased risk of death in patients with the lowest zinc levels (<579 μg/L) in comparison to patients with the highest levels (>688 μg/L): HR (hazard ratio) = 2.32; CI (confidence interval) = 1.47–3.69; *p*-value < 0.01.

Table 3 summarizes the multivariable hazard ratios (HR) and associated confidence intervals for various factors on overall survival. Only the relationship between cancer stage (*p* < 0.01) and zinc levels (*p* < 0.01) remained significant in the multivariate model. Compared to tertile C (reference, highest zinc serum level), the HR for tertile A (lowest zinc level) was 1.95 (CI = 1.21–3.17).

Polymorphisms of antioxidant genes by themselves were not correlated with survival using the multivariate model. However the zinc influence on survival was different depending on the genotypes of the antioxidant enzymes. The polymorphism *GPX1 TC/TT* was present in 128 (42.6%) and *GPX1 CC* in 172 (57.4%) patients. There was no statistically significant difference in zinc impact on survival between these polymorphism subgroups. For both variants the risk of death was increased in patients with the lowest zinc levels in comparison to patients with the highest levels: HR = 2.32 (CI = 1.08–5.01; *p* = 0.03) for *GPX1 TC/TT* and HR = 1.93 (CI = 1.01–3.71; *p* = 0.047) for the *GPX1 CC* variant (Table 4). From these results it appears the *GPX1* polymorphism does not influence the effect of zinc on the survival of patients with laryngeal cancer.

With respect to the *SOD2* genotypes, only for the subgroup of *SOD2 TC/TT* patients (219 patients, 73%) was the influence of serum zinc levels on survival statistically significant. For *SOD2 TC/TT* variant carriers the risk of death was increased in patients with the lowest zinc levels in comparison with patients with the highest levels: HR = 3.0; CI = 1.63–5.55; *p*-value < 0.01 and for *SOD2 CC* variant there was no statistically significant difference: HR = 0.83; CI = 0.33–2.14; *p*-value = 0.70 (Table 5).

In subgroup of *CAT CC* patients (162 patients, 54%) the influence of serum zinc levels on survival was stronger and statistically significant compared to *CAT TC/TT* patients. For the *CAT CC* variant the risk of death was increased in patients with the lowest zinc levels in comparison with patients with the highest levels: HR = 2.55; CI = 1.32–4.97; *p*-value < 0.01 and for *CAT TC/TT* variant there was no statistically significant difference (HR = 1.71; CI = 0.85–3.44; *p*-value = 0.129) (Table 6).

Taking into account the results presented in Table 4, Table 5 and Table 6, the influence of zinc on survival is strongly influenced for patients carrying the *SOD2 TC/TT* and *CAT* CC variants. Given only those variants, the serum zinc level impact on the risk of death was stronger compared with the entire cohort. For a subgroup of patients with at least one of the variants present (*SOD2 TC/TT* or *CAT CC* (260 patients, 86%)) the HR with the lowest zinc levels group (A) was 2.5 (CI = 1.48–4.21, *p* < 0.01) compared with those patients from the highest zinc level group (C) (Table 7).

Impact of serum zinc levels on the risk of death in laryngeal cancer patients was multiplied in the subgroup with a combined positive antioxidant enzymes polymorphism profile resulting in a positive influence on survival. In the subgroup combining polymorphisms of *SOD2 TC/TT* and *CAT CC* (121 patients, 40.3%) the HR for the lowest zinc level tertile (A) was 3.13 (CI = 1.32–7.46; *p*-value = 0.01) compared with highest zinc level tertile (C) (Table 8).

On the other hand, in the subgroup of patients in whom neither the *SOD2 TC/TT* nor *CAT CC* variant was found, the effect of zinc on survival was the opposite. The HR for the lowest zinc level tertile (A) was 0.40 (CI = 0.08–1.98; *p*-value = 0.266) compared with highest zinc level tertile (C); however, due to the small size of the group (*n* = 40), the difference was not statistically significant (Table 9).

## 4. Discussion

The results from this study suggest that low levels of serum zinc at the time of diagnosis are associated with a significantly increased risk of death among laryngeal cancer patients who reside in Szczecin, Poland. The effect appeared to be continuous; that is, the risk of death declined continuously with increasing circulating levels of zinc and there was no apparent threshold value. Individuals in the lowest tertile of zinc had a 1.95-fold increased risk of dying during their follow-up period.

Many reports exist regarding the zinc levels in cancer versus the corresponding normal tissues. Decreased levels of zinc in malignant tissues have been shown for pancreatic, hepatocellular, prostate, lung, colon, thyroid, kidney, gall bladder and ovarian cancers [27,28,29,30,31,32,33,34,35]. Decreased levels of zinc in cancers have been shown in malignant tissue and in other specimens such as serum, hair and nails. Analysis of the serum zinc levels has revealed a tendency toward an increased risk of breast cancer for unselected breast cancers and among BRCA1 carriers with low zinc levels [36].

There have been few studies evaluating the correlation between zinc levels and the occurrence of head and neck cancers. Abdulla et al. have shown that zinc in plasma and whole blood from 13 patients with squamous cell carcinoma of the head and neck was significantly lower than in healthy controls [37]. Wozniak reported decreased levels of zinc in hair and nails in a group of 59 patients with larynx, salivary gland, oral cavity and tongue cancers [38]. A study by Varghese revealed that there was a significant reduction in the serum zinc levels in both oral cavity leucoplakia and cancer in the cohort of 100 patients [39]. Other authors have published contrary results. Most recently, a study by Golasik, on the Polish population, revealed a decreased level of zinc in hair but an increase in serum in 66 laryngeal cancer patients compared with healthy controls [40]. Similar observations on increased levels of serum zinc in cancer patients, among them head and neck malignancies, was reported by Pasha [41].

Several studies have examined zinc status and cancer mortality. Data from a French cohort study and the National Health and Nutrition Examination Survey (NHANES) have linked lower zinc levels with increased overall cancer mortality [42]. Similar results have been shown for the US population [43]. In contrast it was reported that serum zinc levels may be not associated with hepatocellular cancer survival [44]. Until now, there has been very limited evidence for a role of zinc status on laryngeal cancer progression or outcome. Abdulla reported that serum zinc was significantly lower in head and neck cancer patients who did not respond to therapy and who died within 12 months compared to those who responded to therapy and had a remission within 12–15 months [37]. To our knowledge, there are no studies that have specifically evaluated the relationship between zinc levels and disease prognosis in laryngeal cancer.

The reference level of Zn in serum is 600–1200 µg/L [45]. The prevalence rate of inadequate zinc levels for the general population of Central and Eastern Europe is about 10% [46]. In our cohort, 118 patients had serum zinc levels below the reference level (39.3%). The best prognosis was observed in the tertile with zinc levels above 688 µg/L.

Zinc deficiency may lead to a wide range of metabolic diseases, endocrine disorders, developmental imbalances, neurodegenerative problems, immune deficiencies, inflammatory pathologies, skin diseases and poor wound healing [17,18]. Zinc is reported to induce apoptosis and cytotoxicity in different tumor types including prostate, ovarian, hepatocellular, pancreatic and breast cancers [47,48,49]. The exact mechanisms underlying this synergism and zinc-induced apoptosis are unclear, but zinc homeostasis and p53 functions appear to be key factors for subsequent physiological processes including DNA repair, cell cycle regulation and response to oxidative stress [50]. Intracellular zinc deficiency can enhance DNA damage due to oxidative stress and also blocks critical cellular signals that drive DNA repair and apoptosis. Zinc deficiency results in increased sensitivity to oxidative stress, increased DNA damage and accelerated carcinogenesis [51]. A study by Jayaraman demonstrated that exposure of pancreatic cancer cells to zinc leads to increased protein degradation and enhanced cell death, proposing zinc as a potential therapy in the treatment of pancreatic cancer [52]. Kocdor reported that zinc supplementation induces apoptosis and enhances antitumor efficacy of docetaxel in non-small-cell lung cancer [53]. Other authors suggest that zinc has the potential to be developed as either a primary treatment or as a second line of defense against ovarian cancers that have developed resistance to currently used chemotherapeutics [54].

Several large observational cohort studies have found that dietary zinc intakes are inversely associated with cancer occurrence and all-cause mortality risks. It was shown that the use of zinc supplements was associated with reduced prostate cancer risk [55]. The Vitamins And Lifestyle cohort study found that, although long-term use of zinc supplements was not associated with reduced prostate cancer risk, it was associated with a reduced risk for advanced prostate cancer [56]. A study from Sweden clearly showed that high dietary zinc intake was associated with a reduced risk for prostate cancer-specific mortality [57].

To our knowledge no studies have been reported for laryngeal cancer. According to data provided on other cancers and the results of our study, zinc supplementation might improve the prognosis in laryngeal cancer patients. However, it is too early to tell from these observations that the association between serum zinc and survival is causal or if the effect of low zinc can be mitigated by zinc supplementation and future studies are required to determine if zinc has therapeutic benefit in this setting.

In our study we examined the most common polymorphisms of antioxidant genes: (1) *SOD2* 47T/C, rs4880, Val16Ala; (2) *CAT* 262C/T, rs1001179; (3) *GPX1* 599C/T, rs1050450, Pro198Leu. We found that these variants by themselves were not correlated with prognosis of laryngeal cancer patients. However, serum zinc level impact on survival in laryngeal cancer patients was influenced by genotypes of *SOD2* and *CAT*. *SOD2 TC/TT* and *CAT CC* were found to be “positive” variants in which zinc serum levels had the strongest correlation with survival. In subgroups with *SOD2 CC* and *CAT TC/TT* there was no statistically significant correlation between zinc serum level and survival. *GPX1* polymorphism did not change the effect of zinc levels on survival.

*SOD2* 47T/C (rs4880) polymorphism or Val16Ala results in the incorporation of either alanine (C allele) or valine (T allele) in the mitochondrial targeting sequence. Experimental data indicate that the Ala containing *SOD2* is targeted into the mitochondria, whereas the Val form of the protein is partially arrested in the inner mitochondrial membrane [58]. The less efficient form (T) would be associated with higher levels of ROS and greater risk of cancer. In numerous studies, the variant C allele was observed as a risk factor for breast cancer [59,60], prostate cancer [61], gastric cancer [62], lung cancer [63], acoustic neuroma [64], hepatocellular cancer [65] and ovarian cancer [66]. *SOD2* expression can also impact cancer progression. Cases with higher *SOD2* activity of the tumor were associated with worse overall survival in renal cell cancer patients [67] as well as occurrence of distant metastasis and reduced survival in salivary adenoid cystic cancer [68] and gastric cancer [69]. Reduced expression of *SOD2* was correlated with poorer survival of patients with aggressive thyroid or adrenal cancers [70]. Little is known about *SOD2* polymorphism on cancer survival. The CC genotype was shown to be associated with reduced risk of death after treatment for breast cancer [71] and no correlation with prognosis was found for gastric cancer [72]. In our study, TC/TT variant carriers, which theoretically are a less efficient form, were found to be positively correlated with zinc impact on survival.

In the case of *CAT*262C/T (rs1001179) polymorphism, compared with the C allele, the variant T allele has been associated with lower enzyme activity and consequently increased levels of ROS [25,26]. The results of association studies of this polymorphism with cancer risk remain inconsistent and inconclusive. The TT genotype was demonstrated to be a risk factor for breast cancer [26] and prostate cancer in men diagnosed aged <65 [73]. In prostate cancer the TT genotype increased the risk for high-stage disease and metastasis, respectively, implying that this polymorphism may also be a risk factor in tumor progression and metastasis [20]. Studies on breast cancer patients suggest that TT genotype is associated with reduced risk of death [71] or has no impact on survival [74]. In our study, zinc had stronger influence on survival in cases of the more active CC variant.

The presence of the *GPX1* T variant allele contributes to an increased risk of melanoma [75] and meningioma [76]. However, the T variant has also been shown to have a protective role in reducing the occurrence of colorectal adenocarcinoma [77]. In cases of lung and breast cancer, opposing results were shown with the T variant increasing [78,79] as well as decreasing [23,80] the risk. For other malignancies such as gastric, hepatocellular, pancreatic, prostate and bladder cancer no correlation between occurrence risk and polymorphism was found [81,82,83,84,85]. The role of *GPX1* in cancer survival remains uncertain. Most studies suggest that high expression of *GPX1* in tumor tissue is correlated with worse survival. This was shown for lung cancer [86], oral cavity cancer [87] and laryngeal cancer [88]. Other data have shown that expression of *GPX1* has no prognostic significance in head and neck cancers, however in advanced T3/4 tumors the expression was lower compared to T1/2 tumors [89]. Polymorphism in *GPX1* gene had no impact on survival in studies concerning breast [74] and gastric cancer [90]. The influence of the polymorphism itself on the level of *GPX1* activity is not unequivocal, however most authors agree that the T variant (Leu) leads to decreased enzyme activity. According to some authors, the catalytic activity of a protein decreases by 5% with each additional copy of the altered allele [22,23,78,91]. In our study, *GPX1* polymorphism did not change the effect of zinc levels on the survival. In both variants the risk of death was increased in patients with the lowest zinc levels in comparison to patients with the highest levels, however for TC/TT variant carriers (i.e., less efficient form), the correlation with zinc on survival was slightly stronger (HR- 2.32 for TC/TT and 1.93 for CC variant).

Molecular mechanisms that would explain the positive effect of zinc on the survival of patients with laryngeal cancer, only in cases of certain antioxidant enzyme polymorphisms, remain unknown and cannot be explained on the basis of the knowledge available in the literature. Further research is needed in this area. However, the results of our research show that the interaction of enzymatic and non-enzymatic (zinc) antioxidant mechanisms is essential for the survival of patients with laryngeal cancer. It is particularly evident in the subgroup combining both “positive’’ variants, *SOD2 TC/TT* and *CAT CC*, which results in strong dependence between survival and zinc serum level with HR = 3.13. This combination was present in 40% of patients. At least one positive variant was present in 86% of patients with survival dependent on serum zinc level with HR = 2.55. These results suggest that zinc supplementation in those patients could have positive effect on survival. On the other hand, 13% of patients had neither the *SOD2 TC/TT* nor *CAT CC* variant and in that particular subgroup zinc influence on survival seemed to be the opposite, suggesting that those patients should be excluded from zinc supplementation. This result, due to the small size of the group, was not statistically significant and further research on larger, neither the *SOD2 TC/TT* nor *CAT CC*, group is needed to confirm this tendency.

The strengths of our study include the large number of patients identified from one institution and the use of fasting blood samples for zinc measurement. We performed a multivariate analyses taking into account the influence of age, sex, clinical stage, chemotherapy, radiotherapy and pack-years. Despite this, our study was not without limitations. Zinc levels were taken after diagnosis and it is possible that the cancer might have influenced the level. Zinc was measured only once and serum levels tend to fluctuate which may account for minor differences in the population under study. Because of limitations in the data in the Polish vital statistics data base we used all-cause mortality as an endpoint. We expect the majority of the deaths (but not all) in this study are due to laryngeal cancer. By using all-cause mortality, we may have underestimated the effect on laryngeal cancer-specific mortality. The individual subgroups were small and it is difficult to compare the effects of zinc in various subgroups.

## 5. Conclusions

The level of zinc in the serum is an important prognostic factor in laryngeal cancer. The influence of serum zinc levels on survival in laryngeal cancer patients can be multiplied if correlated with adequate antioxidant enzymes polymorphisms: *SOD2 TC/TT* and *CAT CC*. A total of 86% of all laryngeal cancer patients have at least one of those genotypes, suggesting survival is dependent on serum zinc level with a HR of 2.55; 40% have both, with a HR of 3.13. Zinc supplementation in those patients might have positive effect on survival. A total of 13% of patients have neither the *SOD2 TC/TT* nor *CAT CC* variant and in that particular subgroup zinc influence on survival seems to be the opposite, suggesting that those patients should be excluded from zinc supplementation.

## 6. Patent

The described discovery in this paper has been covered by Polish patent application owned by Pomeranian Medical University (P.427370).

## Figures and Tables

**Table 1:** Characteristics of the study population (*n* = 300).

		N (Number of Patients)	%
Sex	M (male)	255	85
	F (female)	45	15
Deceased	Yes	116	38.7
	No	184	61.3
Survival time for participants who died before the end of the study period	mean, (range)	26 months (2–96)	
Survival time for entire cohort	mean, (range)	47.6 months (2–101)	
Age	mean, (range)	61 (41–82)	
Smoking status (pack-years)	mean, (range)	37.2 (0–150)	
Smoking status	Yes	288	96
	No	12	4
Stage	I	69	23
	II	40	13.3
	III	66	22
	IV	125	41.7
Radiotherapy	Yes	135	45
	No	165	55
Chemotherapy	Yes	30	10
	No	270	90
Serum zinc level	mean, (range)	640.43 μg/L (357.76–1317.87)	

**Table 2:** Serum zinc level and risk of death of patients with laryngeal cancer (entire group, *n* = 300).

Tertiles	Serum Zinc Level (µg/L)	Number of Patients	
		Dead	Living
**ZnA**	357.76–578.71	50	49
**ZnB**	579.17–687.98	37	62
**ZnC**	688.54–1317.87	29	73
**Cox Regression**			
ZnB (37 and 62) vs. ZnC (29 and 73) HR = 1.43; CI = 0.88–2.34; *p*-value = 0.145			
ZnA (50 and 49) vs. ZnC (29 and 73) HR = 2.32; CI = 1.47–3.69; *p*-value < 0.01			

**Table 3:** Multivariable Cox regression (entire group, *n* = 300).

Feature		HR	CI (2.5–97.5%)	*p*-Value
*Serum zinc level*	A	1.956	1.21–3.17	*<*0.01
	B	1.344	0.82–2.21	0.245
	C	1.0		
Sex	F	1.0	-	-
	M	0.668	0.4–1.12	0.127
Clinical stage	I	1.0	-	-
	II	2.33	0.93–5.83	0.07
	III	2.23	0.98–5.07	0.056
	IV	7.304	3.28–16.26	*<*0.01
Chemotherapy	Yes	0.802	0.43–1.51	0.494
	No	1.0	-	-
Radiotherapy	Yes	1.197	0.73–1.96	0.473
	No	1.0	-	-
Pack-years		1.011	1–1.02	0.069
Age	≤60	1.0	-	-
	>60	1.37	0.92–2.04	0.122
*GPX1*	TC/TT	0.987	0.67–1.44	0.945
	CC	1.0		
*SOD2*	TC/TT	1.042	0.68–1.59	0.847
	CC	1.0		
*CAT*	TC/TT	1.09	0.74–1.6	0.661
	CC	1.0		

**Table 4:** Impact on survival of zinc serum level depending on *GPX1* polymorphism (multivariable Cox regression).

*GPX1 TC/TT* (n = 128)				*GPX1 CC* (n = 172)		
**Tertiles**	**Number of Patients**		vs.	**Tertiles**	**Number of Patients**	
	**Dead**	**Living**			**Dead**	**Living**
ZnA	28	16		ZnA	22	33
ZnB	16	29		ZnB	21	33
ZnC	10	29		ZnC	19	44
ZnB vs. ZnC HR = 1.13; CI = 0.5–2.59; *p* = 0.76				ZnB vs. ZnC HR = 1.66; CI = 0.88–3.14; *p* = 0.118		
ZnA vs. ZnC HR = 2.32; CI = 1.08–5.01; *p* = 0.03				ZnA vs. ZnC HR = 1.93; CI = 1.01–3.71; *p* = 0.047		

**Table 5:** Impact on survival of zinc serum level depending on *SOD2* polymorphism (multivariable Cox regression).

*SOD2 TC/TT* (n = 219)				*SOD2 CC* (n = 81)		
**Tertiles**	**Number of Patients**		vs.	**Tertiles**	**Number of Patients**	
	**Dead**	**Living**			**Dead**	**Living**
ZnA	42	37		ZnA	8	12
ZnB	26	49		ZnB	11	13
ZnC	16	49		ZnC	13	24
ZnB vs. ZnC HR = 1.60; CI = 0.85–3.03; *p* = 0.144				ZnB vs. ZnC HR = 0.70; CI = 0.28–1.77; *p* = 0.46		
ZnA vs. ZnC HR = 3.0; CI = 1.63–5.55; *p <* 0.01				ZnA vs. ZnC HR = 0.83; CI = 0.33–2.14; *p* = 0.70		

**Table 6:** Impact on survival of zinc serum level depending on *CAT* polymorphism (multivariable Cox regression).

*CAT CC* (n = 162)				*CAT TC/TT* (n = 138)		
**Tertiles**	**Number of Patients**		vs.	**Tertiles**	**Number of Patients**	
	**Dead**	**Living**			**Dead**	**Living**
ZnA	24	25		ZnA	24	26
ZnB	17	41		ZnB	21	20
ZnC	15	40		ZnC	14	33
ZnB vs. ZnC HR = 1.15; CI = 0.56–2.39; *p* = 0.692				ZnB vs. ZnC HR = 1.75; CI = 0.85–3.64; *p* = 0.129		
ZnA vs. ZnC HR= 2.55; CI = 1.32–4.97; *p* < 0.01				ZnA vs. ZnC HR = 1.71; CI = 0.85–3.44; *p* = 0.129		

**Table 7:** Multivariable Cox regression (SOD_2_ *TC/TT* or *CAT CC*, n = 260).

Feature		HR	CI (2.5–97.5%)	*p*-Value
***Serum zinc level***	A	2.497	1.48–4.21	*<*0.01
	B	1.318	0.76–2.28	0.324
	C	1.0		
Sex	F	1.0		
	M	0.72	0.42–1.24	0.238
Clinical stage	I	1.0		
	II	2.379	0.9–6.29	0.08
	III	2.207	0.93–5.25	0.073
	IV	5.916	2.54–13.77	*<*0.01
Chemotherapy	Yes	0.792	0.41–1.53	0.488
	No	1.0		
Radiotherapy	Yes	1.628	0.96–2.76	0.071
	No	1.0		
Pack-years		1.009	1–1.02	0.147
Age	≤60	1.0		
	>60	1.205	0.79–1.84	0.39

**Table 8:** Multivariable Cox regression (*SOD2 TC/TT* and *CAT CC*, n = 121).

Feature		HR	CI (2.5–97.5%)	*p*-Value
***Serum zinc level***	A	3.134	1.32–7.46	0.01
	B	1.446	0.57–3.69	0.44
	C	1.0		
Sex	F	1.0		
	M	0.707	0.31–1.63	0.415
Clinical stage	I	1.0		
	II	3.812	0.82–17.81	0.089
	III	3.178	0.75–13.51	0.117
	IV	15.246	3.83–60.72	*<*0.01
Chemotherapy	Yes	0.238	0.07–0.84	0.026
	No	1.0		
Radiotherapy	Yes	1.33	0.59–2.98	0.49
	No	1.0		
Pack-years		1.003	0.98–1.02	0.718
Age	≤60	1.0		
	>60	1.11	0.57–2.17	0.761

**Table 9:** Multivariable Cox regression (without *SOD2 TC/TT* and without *CAT CC*, n = 40).

Feature		HR	CI (2.5–97.5%)	*p*-Value
***Serum zinc level***	A	0.409	0.08–1.98	0.266
	B	0.447	0.07–2.88	0.397
	C	1.0		
Sex	F	1.0		
	M	0.291	0.03–2.74	0.28
Clinical stage	I	1.0		
	II	3.752	0.21–67.48	0.37
	III	1.212	0.07–20.82	0.894
	IV	142.487	6.16–3293.38	*<*0.01
Chemotherapy	Yes	0.826	0.08–8.92	0.875
	No	1.0		
Radiotherapy	Yes	0.061	0.01–0.44	*<*0.01
	No	1.0		
Pack-years		1.056	1.01–1.1	*<*0.01
Age	≤60	1.0		
	>60	2.958	0.58–15.15	0.193

## Data Availability

Data supporting reported results are available from the first author to all interested researchers.
